# Intervention for Intraoperative Teaching in Anesthesiology Using Weekly Keyword Program: Development and Usability Study

**DOI:** 10.2196/42060

**Published:** 2023-05-18

**Authors:** George Tewfik, Rotem Naftalovich, Carlos Rodriguez-Aponte, Bishoy Ezzat

**Affiliations:** 1 Rutgers New Jersey Medical School Newark, NJ United States

**Keywords:** resident teaching, intraoperative teaching, educational strategies, teaching, anesthesiology, education, efficacy, survey, electronic, medical residents, operation

## Abstract

**Background:**

Learning in the operating room (OR) for residents in anesthesiology is difficult but essential for successful resident education. Numerous approaches have been attempted in the past to varying degrees of success, with efficacy often judged afterward using surveys distributed to participants. The OR presents a particularly complex set of challenges for academic faculty due to the pressures required by concurrent patient care, production pressures, and a noisy environment. Often, educational reviews in ORs are personnel specific, and instruction may or may not take place in this setting, as it is left to the discretion of the parties without regular direction.

**Objective:**

This study aims to determine if a structured intraoperative keyword training program could be used to implement a curriculum to improve teaching in the OR and to facilitate impactful discussion between residents and faculty. A structured curriculum was chosen to allow for the standardization of the educational material to be studied and reviewed by faculty and trainees. Given the reality that educational reviews in the OR tend to be personnel specific and are often focused on the clinical cases of the day, this initiative sought to increase both the time and efficiency of learning interactions between learners and teachers in the stressful environment of the OR.

**Methods:**

The American Board of Anesthesiology keywords from the Open Anesthesia website were used to construct a weekly intraoperative didactic curriculum, which was distributed by email to all residents and faculty. A weekly worksheet from this curriculum included 5 keywords with associated questions for discussion. The residents and faculty were instructed to complete these questions on a weekly basis. After 2 years, an electronic survey was distributed to the residents to evaluate the efficacy of the keyword program.

**Results:**

A total of 19 teaching descriptors were polled for participants prior to and following the use of the intraoperative keyword program to assess the efficacy of the structured curriculum. The survey results showed no improvement in intraoperative teaching based on respondent perception, despite a slight improvement in teaching time, though this was statistically insignificant. The respondents reported some favorable aspects of the program, including the use of a set curriculum, suggesting that greater structure may be beneficial to facilitate more effective intraoperative teaching in anesthesiology.

**Conclusions:**

Although learning is difficult in the OR for residents, the use of a formalized didactic curriculum, centered on daily keywords, does not appear to be a useful solution for residents and faculty. Further efforts are required to improve intraoperative teaching, which is well known to be a difficult endeavor for both teachers and trainees. A structured curriculum may be used to augment other educational modalities to improve the overall intraoperative teaching for anesthesia residents.

## Introduction

Education is a critical component of residency training; however, learning in the operating room (OR) for residents in anesthesiology is difficult and unstructured [1]. Various techniques have been used in the past to improve intraoperative teaching in anesthesiology. Faculty in residency programs have attempted to educate residents using traditional lectures on set topics, problem-based learning discussions, or case debriefing [1-3]. Attempts have been made to improve anesthesia education in the OR using a systematic approach to curriculum development and clearly defining study topics. Walsh et al [1] used a stepwise progression from a generalized needs assessment to a targeted needs assessment, defining goals and objectives and using various educational strategies and implementation.

Anesthesia education efficacy is difficult to assess due to the subjective nature of teaching and the rare measurable data points of formal examinations. Survey-based assessments to ascertain efficacy have been deployed in the past. Wakatsuki et al [4] used this methodology to conclude that in teaching, incorporating autonomy, reasoning, literature, prior knowledge, flexibility, reflection, as well as real-time feedback and teach back are most efficacious.

An important consideration when discussing intraoperative teaching is the maintenance of safety in patient care and vigilance for ongoing procedures. The practice of reading intraoperatively during periods of maintenance anesthesia [5] has been observed to have no significant effect on vigilance or responsiveness to adverse events. Other perceived barriers to successful intraoperative education include clinical production pressure on anesthesiologists [6].

Intraoperative teaching is also difficult for surgical services, in which faculty and residents spend the majority of their time engaging in patient care. A surgical study by Iwaszkiewicz et al [7] showed that faculty efforts to maintain a positive attitude toward teaching, establishing a calm and courteous environment, and providing “hands on” learning for residents contributed to improved perceptions by residents regarding intraoperative teaching [7]. Past studies have shown that acute stress is nearly ubiquitous in surgery and in the OR specifically, affecting both surgical performance and patient safety [8]. Interestingly, more recent studies have shown acute stress in the OR to cause both negative and positive effects on clinical performance [9]. Formalized training for faculty using evidence-based teaching frameworks has also been used with success [10]. Moreover, simulation has been used effectively by orthopedic surgical training programs to teach skills to trainees and residents [11].

Another interesting approach to intraoperative education for residents is the “briefing, intraoperative teaching, debriefing” model [12]. This model describes the use of a briefing to identify objectives for the case, intraoperative teaching focused on these objectives, and a debriefing after the case to reflect upon the events that have transpired [12]. Nonetheless, significant barriers have been identified in surgical literature regarding the gap in perception between residents and faculty regarding preparation for intraoperative learning and perioperative feedback, limiting the efficacy of perioperative education [13]. This discordance extends to large differences in the perception between trainees and faculty regarding both the quantity and quality of intraoperative teaching, though Timberlake et al [14] recommend a structured approach to perioperative teaching before, during, and after surgical cases.

## Methods

### Overview

The American Board of Anesthesiology keywords (archived online by the joint Open Anesthesia–International Anesthesia Research Society partnership) were used for a new intraoperative learning curriculum for the Department of Anesthesiology at Rutgers New Jersey Medical School [15].

Each week, 5 keywords were selected at random from the American Board of Anesthesiology keywords list, and a series of questions (3-5 per keyword) distributed based primarily upon the information on the Open Anesthesia website. The questions were open-ended to promote conversation between residents and faculty. The residents were instructed to choose a keyword each day to discuss with their assigned intraoperative faculty and to make that determination the night before so that both faculty and residents could study the topic ahead of time. Keywords were sent via email to residents and faculty on each Friday for the following week.

The keyword program began in March of 2019, and instructions were given in detail both at the beginning of the program and at regular intervals. The program continued for 2 years prior to evaluation by resident surveys. The survey to assess the efficacy of the keyword program was a modified version of the Anesthesia Theater Education Environment Measure (ATEEM) questionnaire [16]. The ATEEM questionnaire was modified into 19 questions assessing the efficacy of intraoperative teaching [16]. The Likert scale was used, scoring each category 1-5 from “Strongly Agree” to “Strongly Disagree.” Residents were asked to answer these questions comparing and contrasting days in the OR room when the keyword program was used for teaching and days when no keywords were discussed. Several additional questions were also added to the survey to assess the differences in time spent teaching, residents’ perceptions on the most successful overall modalities of intraoperative teaching, and the most effective characteristics of the keyword program for teaching.

Note that despite the distribution of the weekly keywords to all members of the department, keyword discussions did not occur between the residents and faculty daily. This was due to changes in staffing, changes in cases or OR assignments, emergent cases, or an inability of the faculty member to remain in the OR during maintenance anesthesia, when most intraoperative teaching occurs.

### Ethics Approval

Institutional review board of experimental protocols was approved by Rutgers University (reference number Pro2019001411). All methods were carried out in accordance with the relevant guidelines and regulations. All participants signed informed consent for participation in this study. No compensation was provided for the study participants. Moreover, study data were deidentified prior to analysis.

## Results

The program was initiated in July of 2019 and continued for 2 years until June 2021. Surveys were distributed to residents in June 2021, and 54 responses were recorded for the surveys, accounting for 90% of all residents during this time period. The results of the responses to the 19 questions (Textbox 1)—comparing days in which the keyword program was and was not used—were assessed for differences using the paired 2-tailed sample *t* test. No statistically significant changes were found between the 2 groups of responses, indicating no effect for the program. The residents were asked to rate each of these descriptors on the Likert scale for days when keywords were used and for days when keywords were not used for intraoperative teaching. Using an value of .05, no category demonstrated significant difference between the 2 groups.

**Figure 1:**
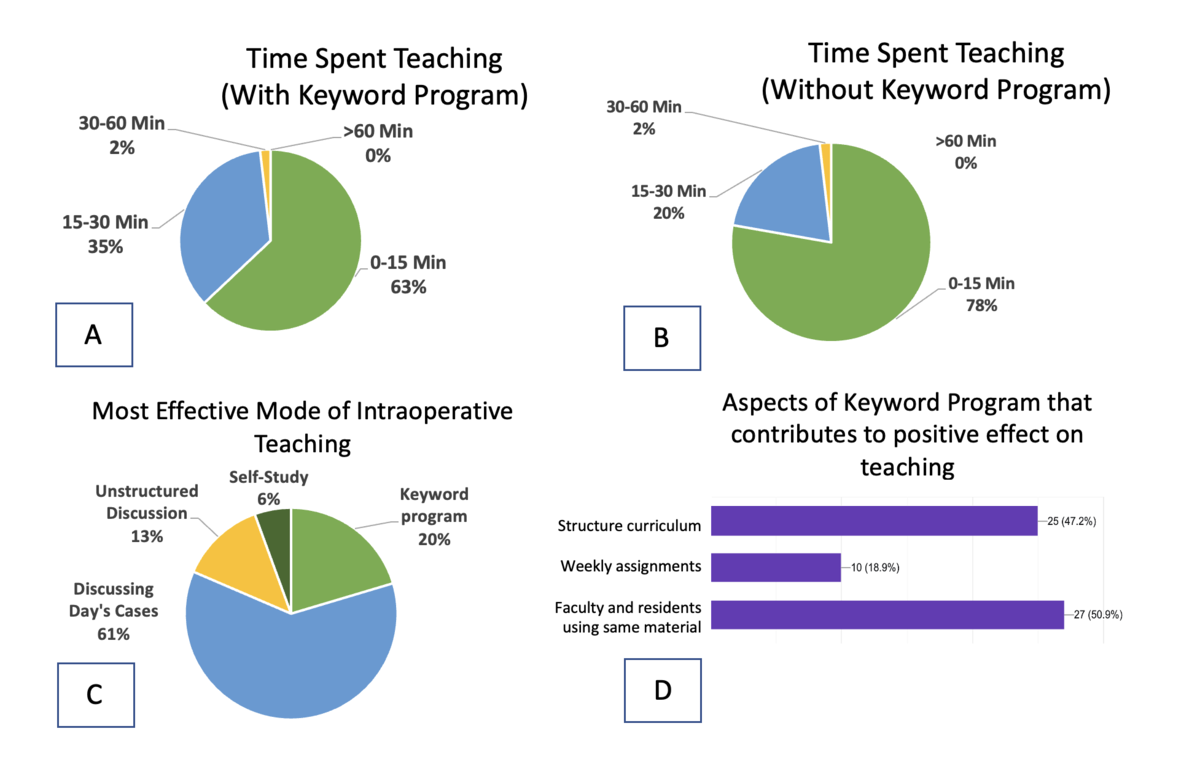
A-B: the differences in time spent teaching on days with or without keywords (though no statistical significance found). 1C: survey results when residents were asked about the most efficacious mode of intra-operative teaching. 1D: respondents’ choices regarding the aspects of the keyword program that they deemed to contribute positively to intra-operative teaching.

Figure 1 includes additional survey results that demonstrate a statistically insignificant increase in time spent teaching, the most effective mode of teaching, and potential aspects of the keyword program found to contribute positively to intraoperative teaching. The respondents who stated that 0-15 minutes a day were spent on resident education decreased from 78% (42/54) to 63% (34/54) when keywords were integrated into the day’s instruction, and they increased from 20% (11/54) to 35% (19/54) for those indicating that 15-30 minutes a day were spent on learning (Figure 1A,B). Only 20% (11/54) of the respondents indicated that the structured keyword program was the most effective tool for intraoperative learning, with 61% (33/54) reporting that the discussion of the current clinical case was more efficacious and conducive to learning (Figure 1C). The aspects of the keyword program that were found to be most helpful for intraoperative learning include using a structured curriculum (25/53, 47.2%) and using the same curriculum for faculty and residents to study (27/53, 50.9%; Figure 1D).

**Teaching descriptors assessed using the Anesthesia Theater Education Environment Measure tool in the survey form following 2 years of the keyword intraoperative teaching program.**

The teaching helps to develop my confidence.

I receive effective supervision from the clinical teachers.

Teaching is done at appropriate times not affecting vigilance.

I receive teaching anesthetic specialty areas targeted at my learning needs.

The teacher helps to develop my competence.

My clinical teachers are accessible for advice.

I experience friendly relations with my teachers in the operating room.

The clinical teachers in this hospital interact well with trainees.

My clinical teachers promote an atmosphere of mutual respect.

I have an appropriate level of clinical responsibility.

My clinical teachers are clear in their teaching.

I am clear about the learning objectives of teaching sessions in the operating room.

I receive the necessary clinical supervision.

I have a good collaboration with anesthesia staff.

I have the opportunity for on-the-job learning.

My clinical teachers have established good rapport with me.

I am encouraged to participate in the theatre setting.

There is a systematic clinical training program.

I feel able to ask the question I want.

## Discussion

### Principal Findings

This investigation sought to improve intraoperative teaching by incorporating a structured curriculum with keywords for residents in anesthesiology and the faculty. Unfortunately, there was no demonstrable positive effect to this intervention. There was essentially no statistically significant difference in the responses by the residents to a survey when comparing intraoperative teaching with and without the use of keywords. This may demonstrate a failure on the part of the initiative to make meaningful improvements to intraoperative teaching. The survey used for the study was an ATEEM tool, which is a validated structure to assess education in anesthesiology intraoperatively, and it was modified to assess the efficacy of the keyword program in this study.

In Figure 1, resident survey respondents noted an overall decrease in time spent during intraoperative teaching when keywords were not used by residents and faculty. The respondents who believed teaching constituted 15-30 minutes of the day fell from 35% (19/54) to 20% (11/54) when keywords were not used, and the category of 0-15 minutes increased from 63% (34/54) to 78% (42/54) in this cohort. Figure 1C shows that resident survey respondents believe that despite the years-long implementation of this keyword program, the most efficacious form of intraoperative teaching is discussing the cases of the day. This may suggest that intraoperative teaching is more effective when didactic material matches the clinical case that is commanding the resident’s attention during the workday.

Nonetheless, the residents identified several characteristics of the keyword program that they believe contributed to a positive effect of keywords on intraoperative teaching in Figure 1D, including the fact that keywords forced residents and faculty to use the same educational material simultaneously, and that the keywords used a structured curriculum. These aspects of the keyword program may offer possibilities for future educational interventions to improve the intraoperative instruction of resident trainees.

Intraoperative learning is a notoriously difficult task for educators of residents in medicine. Past studies have attempted to use such modalities as traditional lectures, problem-based learning discussions, and case debriefing, as well as the targeted assessment of residents [1-3]. Our study attempted to use a set curriculum to teach residents in the OR theater, and to assess the efficacy of this program with a validated survey-based approach [4]. It is unclear exactly why this approach failed, but it is possible that focusing on an unrelated topic during a surgical procedure may not have been feasible due to the aforementioned clinical production pressure, which has been demonstrated to be a barrier to successful intraoperative education [6]. It is also more than likely that any efforts to improve education in the OR have a large barrier to success due to the acute stress caused by the environment [8].

### Limitations

Limitations of this study include a lack of assessment of the percentage of time when the intraoperative keyword curriculum was used by learners. The keywords were sent to both residents and faculty weekly, with no mechanism in place to ensure a successful adherence to the program. This was deemed too difficult due to clinical production pressure, patient emergencies, call burden, vacations, and off-site rotations. Nonetheless, this is a significant limitation, because survey respondents may be included who did not participate in the program or use the keywords in a meaningful manner during the study period.

### Conclusions

After using this intraoperative keyword teaching program for more than 2 years, this study revealed that it had a minimal effect on intraoperative teaching between the anesthesiologist resident trainees and faculty. Consideration should be given to alternate methodologies to improve intraoperative teaching for learners in the anesthesiology residency. The results presented in this study may suggest characteristics of a future intervention that may be more successful in improving intraoperative education. Nearly half of the respondents agreed that the use of a structured curriculum contributed positively to education. Perhaps the use of a structured curriculum that is custom tailored to a resident’s current rotation (instead of an arbitrary schedule, which was employed in this study) may be more efficacious for residents’ educational enhancement. It was also noted by the survey respondents that the coordination of educational material between the residents and faculty was advantageous for learning. This could be incorporated into a policy in which the resident and faculty member plan their educational discussions ahead of time to provide both the teacher and the learner the opportunity to review a chosen topic before their review together in the OR.

## Abbreviations

ATEEM: Anesthesia Theater Education Environment Measure
OR: operating room

## Data Availability

All data generated or analyzed during this study are included in this article.
